# Evolution of Human Memory B Cells From Childhood to Old Age

**DOI:** 10.3389/fimmu.2021.690534

**Published:** 2021-07-23

**Authors:** Michela Ciocca, Salvatore Zaffina, Ane Fernandez Salinas, Chiara Bocci, Patrizia Palomba, Maria Giulia Conti, Sara Terreri, Giuseppe Frisullo, Ezio Giorda, Marco Scarsella, Rita Brugaletta, Maria Rosaria Vinci, Nicola Magnavita, Rita Carsetti, Eva Piano Mortari

**Affiliations:** ^1^ Diagnostic Immunology Research Unit, Multimodal Medicine Research Area, Bambino Gesù Children’s Hospital, IRCCS, Rome, Italy; ^2^ Occupational Medicine/Health Technology Assessment and Safety Research Unit, Clinical-Technological Innovations Research Area, Bambino Gesù Children’s Hospital, IRCCS, Rome, Italy; ^3^ Health Directorate, Occupational Medicine, Bambino Gesù Children’s Hospital, IRCCS, Rome, Italy; ^4^ Department of Molecular Medicine, Sapienza University of Rome, Rome, Italy; ^5^ Diagnostic Immunology Clinical Unit, Department of Diagnostic and Laboratory Medicine, Bambino Gesù Children’s Hospital, IRCCS, Rome, Italy; ^6^ Department of Maternal and Child Health, Policlinico Umberto I, Sapienza University of Rome, Rome, Italy; ^7^ Core Facilities, Bambino Gesù Children’s Hospital, IRCCS, Rome, Italy; ^8^ Post-Graduate School of Occupational Health, Section of Occupational Medicine and Labor Law, Università Cattolica del Sacro Cuore, Rome, Italy; ^9^ Department of Woman, Child & Public Health, Fondazione Policlinico Universitario A. Gemelli IRCCS, Rome, Italy

**Keywords:** B cell, CD27, T cell-independent B-cell activation, aging, memory B cell (MBC)

## Abstract

High quality medical assistance and preventive strategies, including pursuing a healthy lifestyle, result in a progressively growing percentage of older people. The population and workforce is aging in all countries of the world. It is widely recognized that older individuals show an increased susceptibility to infections and a reduced response to vaccination suggesting that the aged immune system is less able to react and consequently protect the organism. The SARS-CoV-2 pandemic is dramatically showing us that the organism reacts to novel pathogens in an age-dependent manner. The decline of the immune system observed in aging remains unclear. We aimed to understand the role of B cells. We analyzed peripheral blood from children (4-18 years); young people (23-60 years) and elderly people (65-91 years) by flow cytometry. We also measured antibody secretion by ELISA following a T-independent stimulation. Here we show that the elderly have a significant reduction of CD27^dull^ memory B cells, a population that bridges innate and adaptive immune functions. In older people, memory B cells are mostly high specialized antigen-selected CD27^bright^. Moreover, after *in vitro* stimulation with CpG, B cells from older individuals produced significantly fewer IgM and IgA antibodies compared to younger individuals. Aging is a complex process characterized by a functional decline in multiple physiological systems. The immune system of older people is well equipped to react to often encountered antigens but has a low ability to respond to new pathogens.

## Introduction

The progressive deterioration of the immune system in aged individuals is termed “immunosenescence” (1). This process entails an impairment of the immune response with a consequent increased susceptibility to emerging pathogens and a reduced responsiveness to vaccination (2–4). As a consequence, older individuals showed an increased morbidity (4) and mortality (5).

The aging of workers is one of the most important issues for occupational health and safety in Europe. The EU’s demographic old-age dependency ratio (i.e., the ratio between people aged 65 years and over and those aged 20-64) is projected to increase significantly in the coming decades. From about 29% in 2010, it is projected to rise to 59% in 2070 (6). Labor force participation is projected to increase, in particular among older workers, on account of implemented pension reform. Keeping workers active and productive through health promotion intervention is a prime objective of European labor policy (7, 8). This task requires adapting work environments to the changing characteristics of the workforce, taking into account the high prevalence of chronic diseases in older workers and their greater sensitivity to infections (9). The SARS-CoV-2 pandemic is dramatically showing us that the organism reacts to novel pathogens in an age-dependent manner. Disease severity and mortality rate are highest in the elderly, especially when co-existing morbidities further weaken the organism (10, 11) rendering the immune response against the virus less effective (12–14).

With aging, the organism experiences a progressive impairment of both the innate and adaptive arm of the immune system (1), where thymic involution and the decreased output of T cells are probably the most recognized signs (15). Many other immune cell functions are affected by aging, such as reduced antigen-presenting and cytokine secretion ability of macrophages and follicular dendritic cells (16, 17) and diminished neutrophils phagocytic activity (18). In addition, T cell repertoire and T cell responsiveness are reduced (19, 20).

On the other hand, the role of B cells in immune aging remains unclear. Recent findings illustrate major shifts in B cell subsets in the elderly, suggesting that age-related changes in B cells may contribute to immunosenescence (21).

Immunological memory is the ability of our immune system to remember previously encountered pathogens; it is acquired by experience and it changes throughout life affecting our susceptibility to infections. Neonates and children under 5 years are more susceptible to infections compared to adults, because they are continuously exposed to pathogens that they have never encountered before (22). Infections are rare in adults thanks to their large repertoire of specific memory T and B cells generated by previous antigenic experiences. In the elderly, susceptibility to infections increases again (23).

Respiratory tract infections are a major cause of disease during old age (24) when the response to vaccination is decreased (25). Encapsulated bacteria (mostly *Streptococcus pneumoniae*) are the main cause of respiratory tract infections and a leading cause of mortality in people >65 years old (26). As for respiratory infections, also gastrointestinal infectious diseases in the elderly are common (27) suggesting that mucosal immune responses could be compromised (24).

We focused our attention on B cells that change with age in number and type (21). As a result of infection or vaccination, B cells become memory B cells (MBCs) and plasmablasts (PBs) able to produce high affinity antigen-specific antibodies. MBCs can be identified by the expression of the CD27 marker (28). The intensity of expression of CD27 defines two populations, CD27^dull^ and CD27^bright^ MBCs.

CD27^dull^ MBCs can be generated without T cells and germinal center (GC) and are mostly of the IgM isotype. Instead, CD27^bright^ MBCs are generated exclusively in the GC by a T cell-dependent mechanism and under a strong selective pressure by an antigen (Ag). For this reason, the frequency of somatic mutations (SMs) is low in CD27^dull^ MBCs and significantly higher in CD27^bright^ MBCs (28). We have shown that CD27^dull^ MBCs are the precursors of the CD27^bright^ MBCs and that the human MBC pool is organized in large clones with a root of CD27^dull^ MBCs of the IgM isotype, gradually acquiring more SM and finally generating highly mutated MBCs of IgM or switched isotypes. CD27^bright^ MBC are closer to the plasma cell stage than CD27^dull^ MBCs. In a previous study, we sorted CD27^dull^ and CD27^bright^ MBCs and demonstrated that upon stimulation CD27^bright^ MBCs undergo rapid and extensive proliferation and differentiate in large number of plasmablasts more effectively than CD27^dull^ MBCs (28).

The reduction of CD27^dull^ IgM MBCs correlates with an impaired immune response to encapsulated bacteria in infants, splenectomized and asplenic individuals, and patients with primary or secondary immune deficiency (29). With age, IgM MBCs decrease (30). Moreover, aging has an impact on the splenic marginal zone (MZ) that is known to be the site where CD27^dull^ IgM MBCs (31, 32) are located.

Here we show that, compared to children, elderly individuals have more CD27^bright^ MBCs, suggesting that their immune system may be equipped to react against well-known antigens but has a reduced ability to respond to new pathogens.

Moreover, after *in vitro* stimulation with CpG, B cells from elderly individuals produced significantly fewer IgM and IgA antibodies compared to younger individuals.

## Materials and Methods

### Ethical Approval

Ethical approval was obtained from the Ethics Committee at the Bambino Gesù Children Hospital. According to the guidelines on Italian observational studies as established by the Italian legislation about the obligatory occupational surveillance and privacy management, health care workers’ confidentiality was safeguarded and informed consent was obtained from all the participants. The study was performed in accordance with the Good Clinical Practice guidelines, the International Conference on Harmonization guidelines, and the most recent version of the Declaration of Helsinki.

### Human Samples

We analyzed peripheral blood from children (4-18 years); young people (23-60 years) and elderly people (65-91 years). Young and elderly individuals were health care workers from our hospital, the majority of whom were active, while others were already retired. Older individuals were screened by interview to ensure they did not have autoimmune diseases, tumors, or recent infections.

At the time of blood sampling, none of the subjects had an acute infection or were taking any medication known to alter immune function (such as steroids or statins).

### Cell Isolation

Heparinized peripheral blood was collected and then centrifuged through a Histopaque gradient (Ficoll Paque™ Plus 206, Amersham Pharmacia Biotech) for 25 min at 2400 rpm. PBMCs were collected from the interface. Next, PBMCs were spun down and washed twice with RPMI 1640 medium (Euroclone). Then PBMCs were frozen and stored in liquid nitrogen until use. The freezing medium contained 90% Fetal Bovine Serum (FBS) and 10% DMSO.

### Stimulation and Reagents

PBMCs were loaded with Carboxyfluorescein succinimidyl ester (CFSE, Life Technologies). Briefly, 1x10^6^ cells/ml were resuspended in PBS (Euroclone) with 1% FBS (Gibco BRL) and loaded with 1μM CFSE for 20 min at 37°C. After washing, cells (at a concentration of 5x10^6^ cells/ml) were stimulated with 0.35 μM of TLR9 agonist CpG-B ODN2006 (Hycult Biotech) in complete medium for 5 days at 37°C. Complete medium was prepared as follows: RPMI-1640 (Euroclone), 10% heat inactivated fetal bovine serum (FBS, Hyclone Laboratories), 1% L-Glutammine (Gibco BRL); 1% Penicillin/Streptomicin 100X (Euroclone), 1% sodium pyruvate (Gibco BRL).

### ELISA Immunoassay

Following CpG stimulation (5 days), we performed an ELISA to detect secreted immunoglobulin in the supernatant of the stimulated cells. Briefly, 96-well plates (Costar3590 EIA/RIA plate) were coated overnight with purified goat anti-human IgA+IgG+IgM (H+L chain; Jackson ImmunoResearch Laboratories). After washing with PBS containing 0.05% Tween and blocking with PBS containing 1% gelatin (1 h, room temperature), plates were incubated for 1 h at 37°C with the supernatants of cultured cells. After removing the supernatants and washing, plates were incubated for 1 h with peroxidase-conjugated fragment goat anti-human IgA or IgG or IgM antibodies (Jackson ImmunoResearch Laboratories). The assay was developed with O-phenylendiamine tablets (Sigma-Aldrich) as a chromogenic substrate. Absorbance at 450 nm was measured, and immunoglobulin concentrations were calculated by interpolation with the standard curve.

### Flow Cytometry and Antibodies

A total of 50 ul of total peripheral blood was used for enumeration of CD45+ cells using the DuraClone IM Count (Beckman Counter) following the manufacturer’s instructions. PBMCs were stained with the appropriate combination of fluorochrome-conjugated antibodies to identify B cell subsets according to standard techniques. Used antibodies were: CD19, CD21, CD24, CD27, CD38, IgG, IgA, and IgM (**Table S1**). Acquisition was performed on a BD LSRFortessa X-20 (BD Biosciences) and data were analyzed with FlowJo ver.8 (Treestar). Proliferation was measured through the analysis of CFSE dilution. The FlowJo’s Proliferation tool looks for a pattern typically expressed by cells loaded with fluorescent dye (CFSE in our case) and is allowed to divide. The FlowJo’s Proliferation platform calculates the proliferation index (PI) that corresponds to the mean number of divisions of all cells that have responded (Proliferation Index: Total Number of Divisions/Cells that went into division). We also calculated the replication index (RI) that indicates the fold-expansion of only the responding cells (Replication Index: Total Number of divided cells/The number of cells that went into division). These two indices reflect the intrinsic ability of the different cell types to respond to signals (33).

### Statistical Analysis

Values were compared by the non-parametric Kruskal-Wallis test and, if significant, pairwise comparisons were evaluated by the Mann-Whitney U test nonparametric test. A level of p < 0.05 was considered statistically significant. * p < 0.05; **p < 0.01, and ***p < 0.001.

## Results

### Immunophenotype of B Cells

We determined the frequencies and absolute numbers of B cell populations of children (n=37; 4-18 years) and adults of different ages (20-40 years, n=18);, (65≥ years <70, n=21) and over 70 years (n=9). We excluded infants from our analysis because their memory B cell pool is mainly represented by CD27^dull^ MBCs. Peripheral blood mononuclear cells (PBMCs) were stained with antibodies to CD19, CD21, CD24, CD27, CD38, CD45, and IgM. The gating strategy used to identify B cell population is shown in **Figure S1**. Absolute numbers were calculated by staining blood samples with the DuraClone IM Count (Beckman Counter).

**Figure 1:**
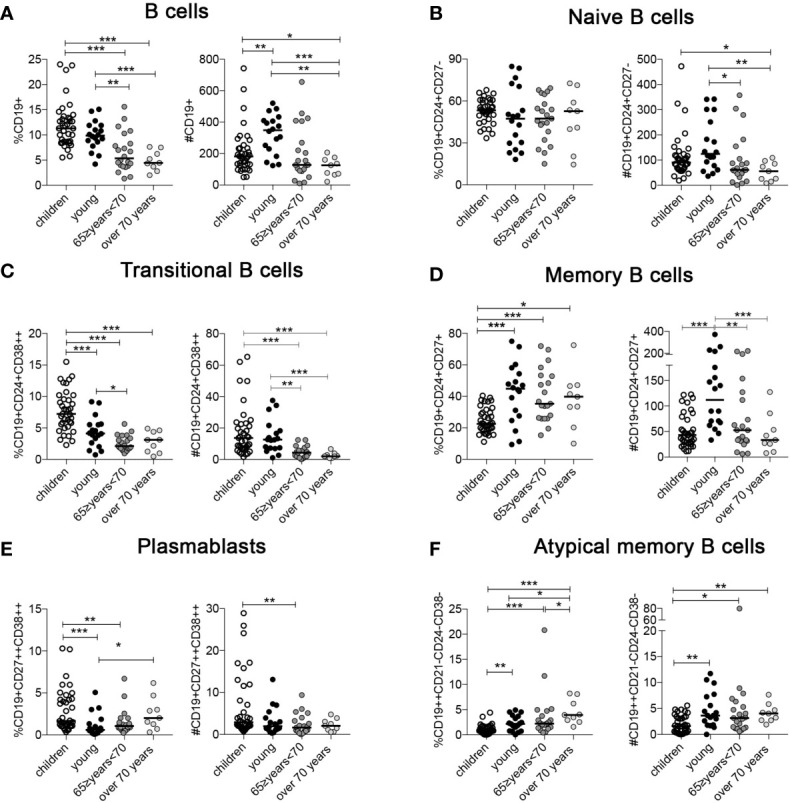
Percentage and absolute numbers of **(A)** B cells; **(B)** naïve B cells; **(C)** transitional B cells; **(D)** memory B cells (MBCs); **(E)** plasmablasts, and **(F)** atypical MBCs (ATM) at the indicated ages (children n = 37; young n = 18; 65 ≥years <70 n = 21; over 70 years n = 9). Midlines indicate the median values. Statistical significances were determined using unpaired, two-tailed Mann-Whitney *U*-tests. *p ≤ 0.05; **p < 0.01; ***p < 0.001.

Children have the highest frequency of B cells in the peripheral blood compared to adults of all ages, (CD19+) (**Figure 1A**) (19). B cells are increased in absolute numbers in young adults probably because of an overall increase in the CD45+ lymphocytes count (**Figure 1A**). The percentages of naïve B cells (CD19+, CD24+, CD27-) appears to be similar in the four groups but absolute numbers indicate a significant decrease in the elderly group probably because of the reduction of total B cells (**Figure 1B**). Transitional B cells (CD19+, CD24+, CD38++), the most immature B cell subtype in the blood, are highest in children and significantly decrease with age, both in percentage and absolute numbers (**Figure 1C**).

The percentage of MBCs (CD19+, CD24+, CD27+) is significantly increased in the young and older adult groups compared to children (**Figure 1D**). As for naive B cells, the total B cell number has a strong impact on MBC absolute numbers and the young group has the highest value compared to the others (**Figure 1D**). In contrast, the percentage of plasmablasts (CD19+, CD27+, CD38++) significantly increased in the over 70 group (**Figure 1E**). Atypical memory B cells (ATM) have been described in aged mice and are distinct from other B cell subsets (34, 35). In humans, they increase in the course of autoimmune diseases (35–37) and infections (38–40), and are thought to reflect a failure or impairment of the germinal center reaction (41). Both percentage and absolute number of ATM (CD19++, CD24-, CD38-, CD21-) were increased in the elderly group (**Figure 1F**) (42).

### CD27^dull^ and CD27^bright^ Memory B Cells

**Figure 2:**
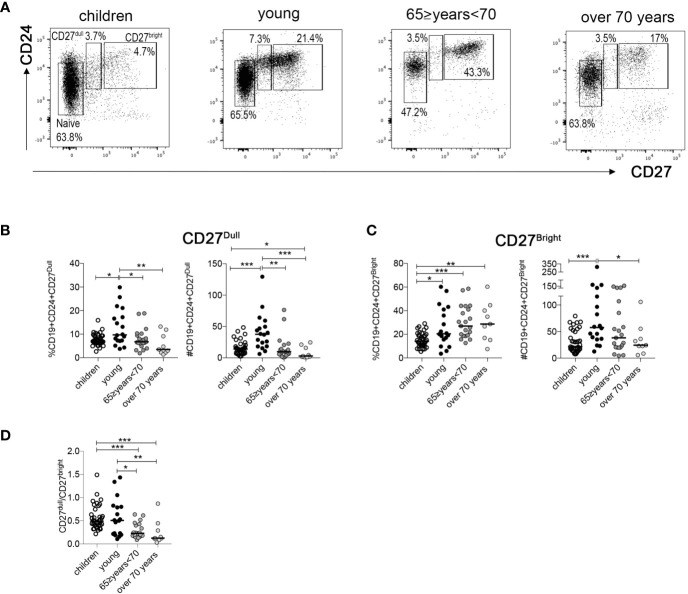
**(A)** Identification of naive, CD27^dull^, and CD27^bright^ MBCs at the indicated ages. **(B, C)** Percentage and absolute numbers of **(B)** CD27^dull^ and **(C)** CD27^bright^ MBCs. **(D)** Ratio of CD27^dull^ and CD27^bright^ MBCs at the indicated ages (children n = 37; young n = 18; 65 ≥years <70 n = 21; over 70 years n = 9). Midlines indicate the median values. Statistical significances were determined using unpaired, two-tailed Mann-Whitney *U*-tests. *p ≤ 0.05; **p < 0.01; ***p < 0.001.

CD27 is the marker that allows the identification of MBCs. Our group have recently demonstrated that MBCs comprise two cell populations, CD27^dull^ and CD27^bright^ MBCs, which are related but have distinct molecular signatures and functions (28). Thus, we examined CD27 intensity in our different age groups. The gating strategy to identify the two MBCs population of each group (gated in CD19+ B cells) is shown in **Figure 2A**. We observed a significant reduction in percentage and absolute number of CD27^dull^ MBCs in the two groups of older people compared to young adults (**Figure 2B**). Instead, the percentage of CD27^bright^ MBCs is significantly lower in the children group compared to the other groups (**Figure 2C**). Interestingly, the absolute number of CD27^bright^ MBCs is significantly increased in the young group, probably because of the higher number of total B cells compared to the other groups (**Figure 2C**).

For our health, it is important to have both CD27^dull^ and CD27^bright^ MBCs since they play different and non-interchangeable roles (28). In order to evaluate the relative size of the two pools, we calculated the ratio between the absolute numbers of CD27^dull^ and CD27^bright^ MBCs at different ages. We found that in the older groups the ratio is significantly lower compared to the two youngest groups (**Figure 2D**), confirming a dramatic reduction of CD27^dull^ compared to CD27^bright^ MBCs.

### CpG Stimulation

The imbalance of B cell frequency is undoubtedly important, nevertheless it is essential to evaluate the ability of B cells to respond to stimuli. Upon T-independent (TI) stimulation with CpG, a ligand of the toll-like receptor 9 (TLR9), B cells proliferate, differentiate into plasmablasts (identified as CD19^low^, CD27++, CD38++), and secrete immunoglobulins (43).

In order to prove their function, PBMCs of children, young and elderly people were cultured for 5 days with CpG and then analyzed by flow cytometry. By ELISA, we measured the amount of IgM, IgG, and IgA in the culture supernatants. Due to the low cell numbers, it was not possible to perform the stimulation on all the samples within each group (19 children, 4 young subjects, and 2 subjects of the 65≥ years <70 were excluded).

**Figure 3:**
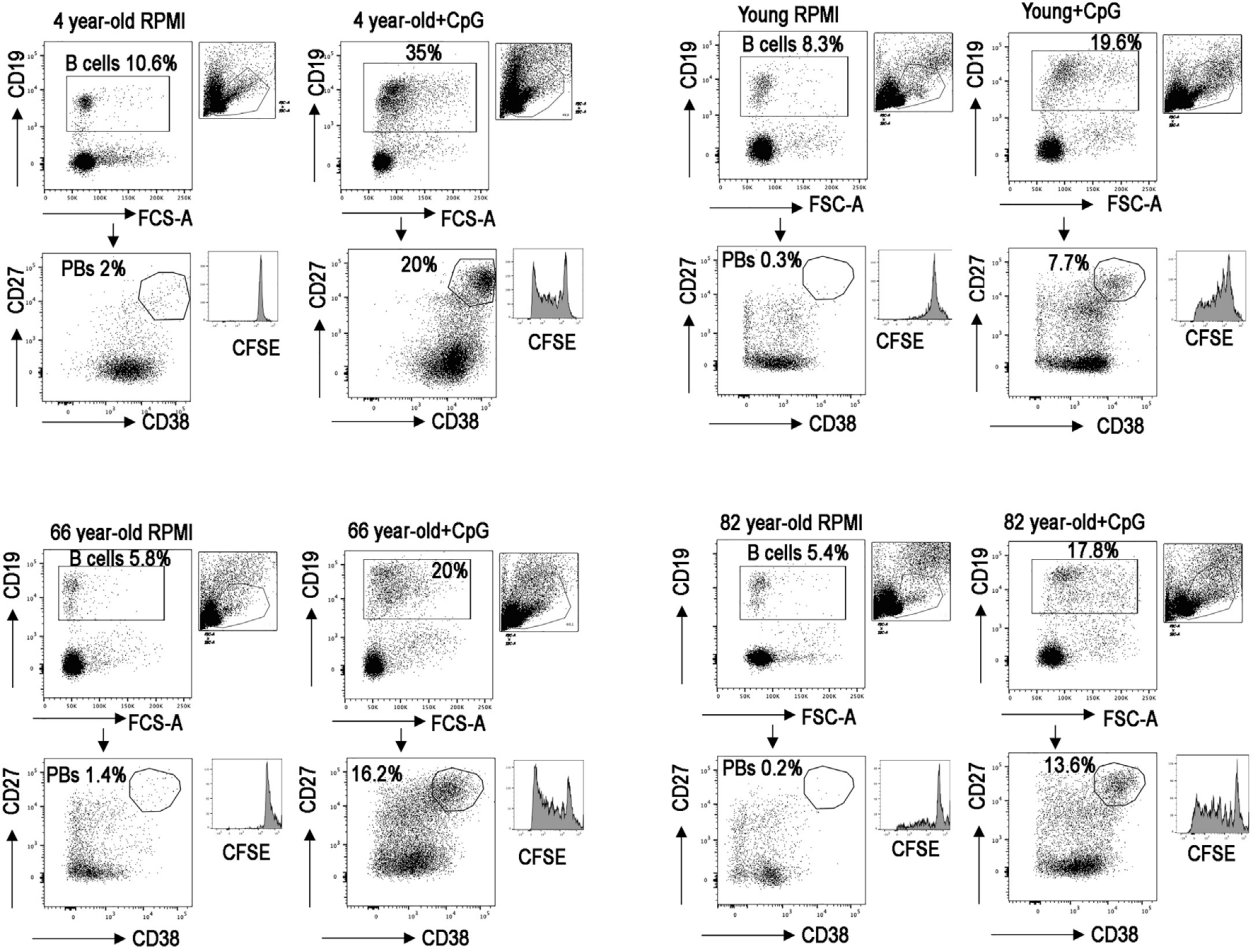
Dot plots show the gating strategy used to identify plasmablasts (PBs), CD19+, CD27++, and CD38++ *in vitro*; in four representative PBMCs, one for each group. Histograms show CFSE.

FACS analysis of four representative samples is reported in **Figure 3**, and the histogram depicts the B cell proliferation evaluated by tracking the dilution of the fluorescent dye CFSE.

**Figure 4:**
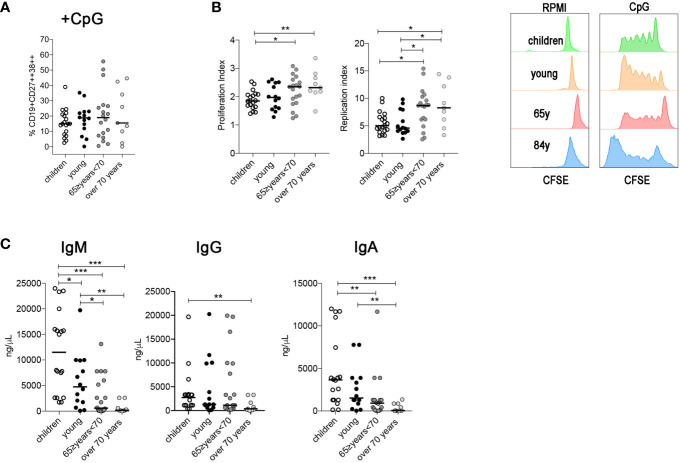
**(A)** Percentage of plasma cells after CpG stimulation (children n = 19; young n = 14; 65≥ years <70 n = 19; over 70 years n = 9). **(B)** Proliferation and replication index gated in B cells after CpG stimulation. **(C)** Plots indicate the concentration of IgM, IgG, and IgA (ng/ml) detected by ELISA in culture supernatants obtained from PBMCs stimulated with CpG. Midlines indicate the median values. Statistical significances were determined using unpaired, two-tailed Mann-Whitney *U*-tests. *p ≤ 0.05; **p < 0.01; ***p < 0.001.

The percentage of *in vitro*-produced plasmablasts was similar between the four groups (**Figure 4A**), suggesting that the ability of MBCs to differentiate in plasmablasts following a TI stimulation is not affected in elderly people. We evaluated also the proliferation and replication index values. The proliferation index is the average number of divisions that all responding cells have undergone since the initiation of the culture and reflects the proliferative capacity of the cells in response to CpG. This index is calculated by dividing the total number of cells that proliferated by the number of cells that went into division.

The replication index determines the fold-expansion of only responding cells and reflects the expansion capacity of the replicating cells rather than the entire culture.

The two indexes were significantly higher in the two oldest groups compared with the children and with the young group (**Figure 4B**).

Although B cells from older groups proliferate and replicate extensively and differentiate into PB, they secrete fewer immunoglobulins. In particular, IgM and IgA levels were significantly reduced in the two groups of older people and the level of IgG was reduced in the over 70 group (**Figure 4C**).

## Discussion

High quality medical assistance and preventive strategies, including pursuing a healthy lifestyle, result in a progressively growing percentage of older people in life and the working environment. It is widely recognized that older individuals show an increased susceptibility to infections and a reduced response to vaccination suggesting that the immune system is less able to react and consequently protect the organism. This represents a burden on health care costs and furthermore raises the question of which interventions may guarantee a good quality of life and reduce the risk of avoidable age-related diseases. The current COVID-19 pandemic emergency has strongly demonstrated that older individuals are more fragile when challenged by an unknown pathogen, fostering the need to understand which mechanisms differentiate the aged immune system from that of a young individual.

It has been recently published (44) that the different responses between young and older individuals are due to the different types of cells that prevail in aging in comparison to the younger population.

In order to investigate in detail the age-dependent changes of B cells, we performed a cytofluorimetric and functional study of B cells in the peripheral blood of children and young blood donors and compared them to two groups of older individuals divided according to age (65≥ years <70 or over 70 years). To avoid health–related bias, we enrolled individuals in the study that did not have an associated co-morbidity or previous diseases of the immune or hematopoietic system.

Besides a general age-related reduction in the percentage of CD19+ B cells, we found specific changes in B cell subpopulations. While naïve, transitional, and MBCs were reduced in either or both percentages and total numbers, we observed an increase in both the percentage and the absolute number of ATM B cells in older individuals compared to young. These cells have been identified in different contexts that include chronic infection, autoimmunity, and aging (45).

CD27^dull^ MBCs in older people were significantly reduced both in percentage and absolute number, as well as the ratio between CD27^dull^ and CD27^bright^. We have previously demonstrated that CD27^dull^ MBCs are mostly of the IgM isotype and can be generated without T cells and GC (28). CD27^dull^ MBCs are the precursors of CD27^bright^ MBCs. While CD27^bright^ MBCs produce antibodies that are highly mutated, CD27^dull^ MBCs are less mutated and may enter the GC reaction in competition with naïve B cells, having the advantage of a more suited transcriptome compared to naïve B cells (28). Thus the reduction of CD27^dull^ MBCs not only explains the reduction of IgM MBCs observed in the elderly (21) but also explains the diminished ability of elderly people to react to new infections and vaccinations associated with the increased incidence and severity of infections (46).

The dramatic reduction of CD27^dull^ MBCs in older individuals can explain the reduction of TI immune responses, such as those against *Streptococcus pneumoniae* (29). Interestingly, the susceptibility to pneumococcal infection is high in neonates whose CD27^dull^ MBCs population is still scarce, reduced in the young and adult populations, where CD27^dull^ MBCs are abundant, and increased again in the elderly (46).

The significant reduction of CD27^dull^ MBCs also has an impact on sIgA synthesis. We have recently demonstrated that IgM MBCs are necessary for the production of sIgA at the mucosal surface (47). sIgA is the major player for host defense, providing a first line of effective immunity against pathogens at mucosal sites. The gastrointestinal tract in the elderly is particularly susceptible to infectious diseases, suggesting that poor mucosal immunity is responsible for the higher mortality to infections in aged individuals (48). Interestingly, it has been shown that at older ages there is a failure of induction of the sIgA antibody resulting in age-related decline in mucosal immunity (49).

B cells fight viruses and bacteria by producing antibodies following their differentiation into circulating plasmablasts. The percentage of plasmablasts increased with age and the ability of the B cells to proliferate and differentiate into plasmablasts seems to not be affected or even improved in older subjects because of the increase of CD27^bright^ MBCs that are closer to the plasma cell stage than CD27^dull^ MBCs (28). However, our results from the *in vitro* studies indicate an impairment of the B cells’ function following a TI stimulus and confirm that the most affected antibodies are IgM and IgA.

Thus, the observed plasmablasts number associated with a reduced level of secreted antibodies suggest an impaired capacity to exert their function with age.

Aging is associated with the accumulation of damaged mitochondria, that are less efficient in ATP generation (50). Several studies indicate that autophagy-related (ATG) proteins and other proteins required for autophagy induction, such as Sirtuin 1, are less expressed in aged tissues and that autophagy diminishes with aging (51). Atg5^-/-^ murine plasma cells show augmented endoplasmic reticulum stress and cell death, failing to give rise to an efficient antibody response and to form long-lived plasma cells *in vivo* (52). Atg5^f/f^ CD19-Cre mice have reduced IgM and IgG responses in both TI and T-dependent immunization experiments, revealing a key adaptive role of autophagy in short-lived plasma cells and in antibodies responses (52).

Taken together these observations suggest that the defects in autophagy observed in the elderly may explain the functional impairment of immunoglobulin secretion by plasmablasts that we have demonstrated in our experiments.

Understanding how the aging of the immune system works is an important step to protect one of the most sensitive groups of our population, especially in this present pandemic era. In some countries, including Italy, governments have issued special emergency regulations that oblige employers to implement special biohazard prevention measures for older workers (53). Among these measures, the provision of vaccines for older workers who are exposed to biohazards is of the utmost importance. Indeed, a recent report highlights novel aging-related genes and adaptive immune dysregulation with potential contributions to the high severity rate of aged COVID-19 patients (54). Thus, vaccination programs may be implemented for individuals that are between 65 and 70 as well as for workers of older age (over 55 years) in order to improve their protection to be used when the natural immune resources start to fail.

The number of subjects in the over 70 years old group is a limitation of our study. Nevertheless, the results are very homogenous and we have been extremely selective during the recruitment. Healthy elderly people represent <15% of the general older population (55) and therefore the subjects who responded to our requirements were rare.

## Data Availability Statement

The raw data supporting the conclusions of this article will be made available by the authors, without undue reservation.

## Ethics Statement

The studies involving human participants were reviewed and approved by Bambino Gesù Children Hospital. Written informed consent to participate in this study was provided by the participants’ legal guardian/next of kin.

## Author Contributions

RC and EP designed the study, analyzed data, and drafted the manuscript. SZ drafted the manuscript. MC, AF, CB, PP, EZ, MS, GF, and ST performed all experiments. RB, MV, and NM collected the samples. MC reviewed the manuscript. All authors contributed to the article and approved the submitted version.

## Funding

This work was funded by the RF2013-02358960 grant from the Italian Ministry of Health.

## Conflict of Interest

The authors declare that the research was conducted in the absence of any commercial or financial relationships that could be construed as a potential conflict of interest.

## Publisher’s Note

All claims expressed in this article are solely those of the authors and do not necessarily represent those of their affiliated organizations, or those of the publisher, the editors and the reviewers. Any product that may be evaluated in this article, or claim that may be made by its manufacturer, is not guaranteed or endorsed by the publisher.

## Supplementary Material

Supplementary Figure 1 Dot plots show the gating strategy used to separate CD19 B cells; CD27^dull^ and CD27^bright^ MBCs and naïve B cells; transitional (Trans) B cells plasmablasts (PBs); and atypical MBCs (ATM) in four representative PBMCs, one for each group.
