# Supplementary figures and images for: Evolution of Human Memory B Cells From Childhood to Old Age
Source: Front Immunol. 2021 Jul 23;12:690534. doi: 10.3389/fimmu.2021.690534 (PMC8343175; doi:10.3389/fimmu.2021.690534)

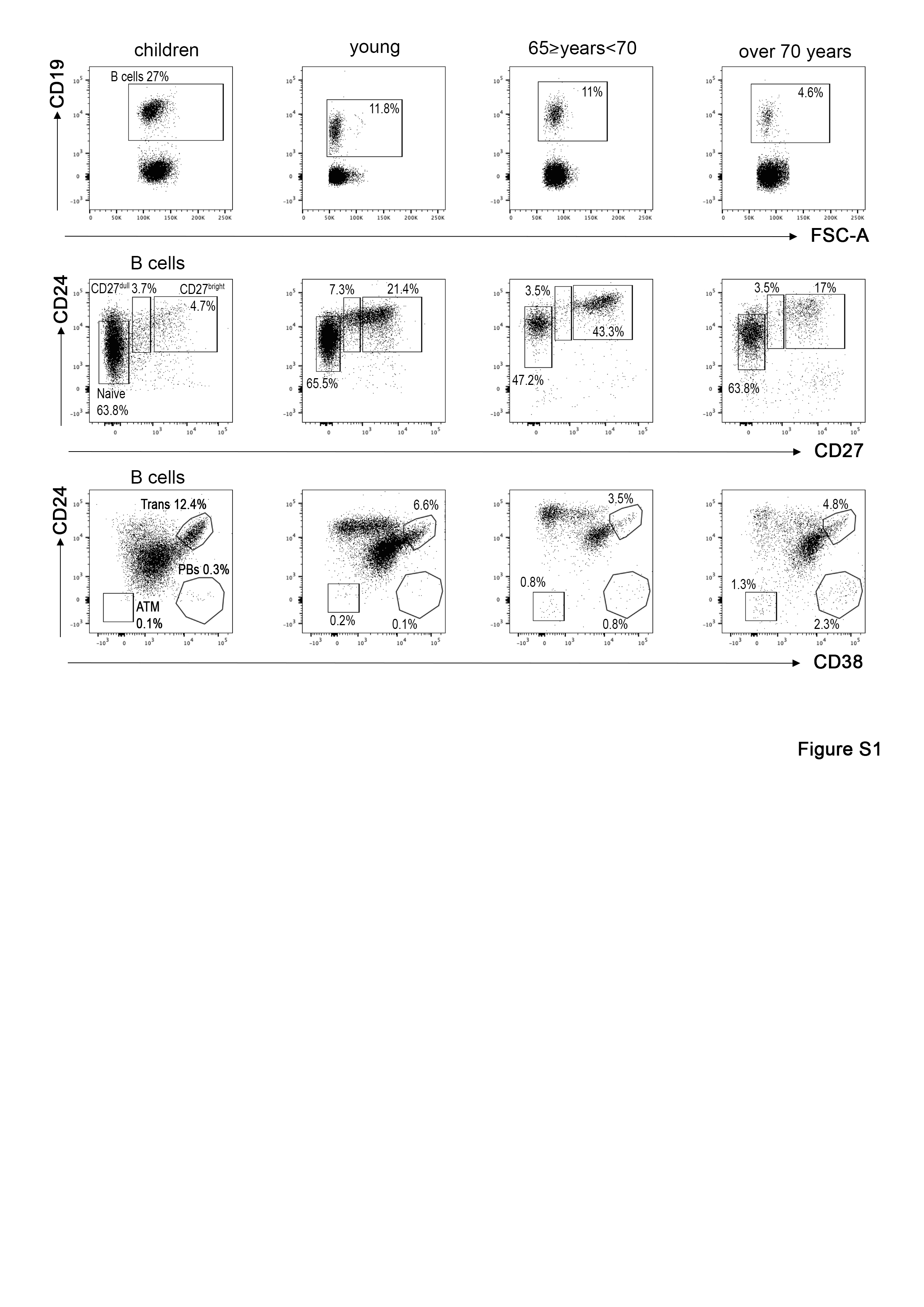

Supplement: Supplementary Figure 1 — Dot plots show the gating strategy used to separate CD19 B cells; CD27dull and CD27bright MBCs and naïve B cells; transitional (Trans) B cells plasmablasts (PBs); and atypical MBCs (ATM) in four representative PBMCs, one for each group. [file Image_1.jpeg]
