# Supplementary material for: Evolution of Human Memory B Cells From Childhood to Old Age
Source: Front Immunol. 2021 Jul 23;12:690534. doi: 10.3389/fimmu.2021.690534 (PMC8343175; doi:10.3389/fimmu.2021.690534)
Supplement: Supplementary file 2 [file Table_1.docx]

**Table S1: Antibodies for staining**

|  | **Clone** | **Catalog number** |
| --- | --- | --- |
| **CD19 BUV737** | SJ25C1 | BD; 612757 |
| **CD24 BV711** | ML5 | BD; 563401 |
| **CD27 PE** | T-271 | BD; 555441 |
| **CD38 BV421** | HIT2 | BD; 562444 |
| **CD21 BV605** | B-ly4 | BD; 740395 |
| **IgG BUV395** | G18-145 | BD; 564229 |
| **IgA FITC** | IS11-8E10 | Miltenyi Biotech; 130-099-107 |
| **IgM APC** | Polyclonal | Jackson ImmunoResearch Laboratories; 709-136-073 |
